# Responding to the increased genetic risk associated with customary consanguineous marriage among minority ethnic populations: lessons from local innovations in England

**DOI:** 10.1007/s12687-016-0269-1

**Published:** 2016-06-16

**Authors:** Sarah Salway, Parveen Ali, Giles Ratcliffe, Elizabeth Such, Nasaim Khan, Helen Kingston, Oliver Quarrell

**Affiliations:** 1 School of Health and Related Research (ScHARR), University of Sheffield, Regent Court, 30 Regent Street, Sheffield, S1 4DA UK; 2 School of Nursing and Midwifery, University of Sheffield, Barber House Annexe, 3a Clarkehouse Road, Sheffield, S10 2LA UK; 3 Health Education Yorkshire and the Humber, Leeds, UK; 4 Manchester Centre for Genomic Medicine, Central Manchester University Hospitals NHS Foundation Trust, Saint Mary’s Hospital, Oxford Road, Manchester, M13 9WL UK; 5 Clinical Genetics Service, Sheffield Children’s NHS Foundation Trust, Western Bank, Sheffield, S10 2TH UK

**Keywords:** Autosomal recessive genetic conditions, Consanguinity, Cousin marriage, Formative service review, Ethnic health inequalities

## Abstract

Populations practising customary consanguineous marriage have a higher incidence of autosomal recessive genetic disorders than those in which reproductive partners are usually unrelated. In the absence of any national-level response, English service developments to address the additional needs of families living with or at risk of such disorders have been locally led. These interventions remain in their infancy here, as elsewhere in Europe, and important questions remain regarding how appropriate, effective and sustainable responses can be operationalised in practice. This formative service review employed four local case studies together with wider consultation exercises over a 4-year period (2011–2015) to document recent responses to this area of need, issues arising and lessons to inform future work. Service components included the following: enhancements to genetic services to provide family-centred, culturally competent approaches to counselling and testing; community genetic literacy approaches; and capacity development among health professionals. Local approaches were, however, very varied in their detail, scope, level of investment and longevity. The provisions of culturally competent genetic counselling services and community-level genetic literacy interventions were generally well received by those who accessed them. Coordinated action across all service components appeared important for an effective service, but healthcare professionals, particularly general practitioners, were often difficult to engage in this agenda. An evaluative culture and engagement in a wider community of practice had supported service development across sites. However, sustaining investment was challenging, particularly where new services were not well integrated into core provision and where commissioning was driven by expectations of short-term reductions in infant mortality and disability.

## Introduction

The practice of marrying close blood relatives, commonly cousins, is a socially acceptable practice in many countries around the world. However, since blood relatives are more likely to carry the same gene variants than unrelated people, populations practising customary consanguineous marriage have a higher incidence of autosomal recessive genetic disorders than those in which reproductive partners are customarily unrelated. Such recessive disorders contribute to increased rates of infant morbidity and mortality. Studies in a variety of settings suggest that the risk of a congenital anomaly is roughly doubled among populations practising close relative marriage (Stoltenberg et al. 1997; Bundey and Aslam 1993). However, accurate estimates of the proportion of conceptions that result in autosomal recessive conditions are compromised by unconfirmed diagnoses and pregnancy terminations. Furthermore, the increased rates documented may also be linked to other risk factors such as higher socio-economic deprivation among couples practising consanguineous marriage (Kerkeni et al. 2007; Bittles 2012). Recent findings from the Born in Bradford cohort in England reported a relative risk of congenital anomaly of 2.19 for offspring of first cousin marriages and 1.99 for less closely related couples when compared to unrelated couples and having controlled for potential confounding factors (Sheridan et al. 2013). In this large study, 2.3 % of all births to unrelated White British couples and 2.6 % of all births to unrelated Pakistani couples had a congenital anomaly compared to 6.2 % of births to first cousin Pakistani couples.

Interest in this issue has grown in recent years in England and other European countries that are home to significant minority ethnic populations of Asian and Arab origin, with debate regarding the appropriate policy and practice response ensuing in the media, community and health policy arenas (Stoltenberg et al. 1997; Department of Health 2012; Hamamy 2012; Teeuw et al. 2014; Shaw and Raz 2015). In the UK, national policy aimed at reducing levels of infant mortality has explicitly recognised the need to address genetic risk associated with consanguinity (Department of Health 2007, 2010, 2012), and the challenge to health and social care services of caring for high numbers of children with severe recessive conditions has been highlighted in some localities (Corry 2002; Morton et al. 2001). The need to improve access to genetic counselling and testing among minority ethnic groups is increasingly acknowledged (DH 2007, 2012; Darr 2010), in part prompted by evidence of low knowledge and poor service uptake among people at risk of recessive conditions (Khan et al. 2010; Darr et al. 2013; Darr et al. 2015).

However, despite growing recognition of the need to address this gap in service provision, to date, no national policy statements, service templates or standards have been produced in the UK. Rather, it is evident that a range of local-level responses are emerging across the country (Darr et al. 2015). Such local-level initiatives appear, to a greater or lesser extent, to be informed by recommendations of the Eastern Mediterranean Regional Office of the World Health Organisation (Alwan and Modell 1997) that have been promoted by UK national experts (Modell and Darr 2002). This approach eschews simplistic attempts to discourage consanguineous marriage at the population level, arguing that such interventions are inappropriate, undesirable and ineffective. Instead, the recommended approach takes a core focus on identifying individuals and families with known genetic risk and improving their access to genetic services, combining three complementary strands of work:

1. Family-centred genetic services for at-risk individuals and families (including identification of index cases, family tracing and proactive offer of counselling and testing).
2. Training to enhance competence and confidence of health professionals, particularly in primary care (to provide information and make appropriate referrals to genetic services).
3. Activities at community level to raise genetic literacy and encourage uptake of services.

To date, there has been only one published evaluation of a UK initiative aimed at addressing increased genetic risk associated with consanguineous marriage. In this case, a multilingual Asian health visitor offered an enhanced genetic counselling and testing service to individuals and families (Khan et al. 2010). The intervention resulted in an increased uptake of genetic counselling and testing services by index couples and their extended family members. A small number of other studies have described service development initiatives and community responses (Nirantharakumar et al. 2010; Qureshi et al. 2003; Darr et al. 2013), but not evaluated their implementation or impact. Importantly also, concerns have been raised about the way in which this area of service need has been framed and responded to. Several commentators have suggested that interventions may risk doing more harm than good, for instance by stigmatising minority groups and/or creating worries about genetic risk without providing any service response (Ahmad 1996; Darr et al. 2015). Clearly, service approaches in this area remain in their infancy in England (as elsewhere in Europe), and important questions remain regarding how culturally appropriate, effective and sustainable responses can be operationalised in practice.

The present paper contributes to our understanding of interventional approaches to addressing increased genetic risk associated with customary consanguineous marriage among minority ethnic groups by reporting on a formative review of service responses in several areas of England. The ongoing design and delivery of a range of contrasting service approaches across England offers the opportunity to extract key lessons that can inform future work. The aims were to describe the content of such initiatives in different areas and to identify factors affecting their development and delivery over time.

## Methods

The current service evaluation was undertaken in support of service development in Sheffield, UK, and employed a longitudinal, pragmatic and formative methodology. In emergent areas of service development—particularly, those that involve complex and socially embedded interventions—a formative approach, that describes intervention components and examines the processes of implementation in practice contexts, can yield important early insight (Craig et al. 2006; Moore et al. 2008).

Data generation was undertaken in three phases and employed a range of qualitative methods and included respondent validation. In phase one in 2011, in consultation with national experts, four localities were identified where initiatives were underway to develop a service response to the issue of genetic risk associated with consanguineous marriage. In all the four localities, a significant proportion of the local population identified themselves as ‘Pakistani/British Pakistani’, and the local infant mortality rate was above the national average. Individuals involved in these initiatives (primarily public health commissioners and genetic service staff) were then contacted and invited to contribute to the learning process. In 2011–2012, a series of complementary data collection activities were undertaken in each site. These included (i) face to face and telephone interviews with staff involved in commissioning or delivering the services (*n* = 12), (ii) a review of relevant documentation including business cases, service specifications, service descriptions, job profiles, health promotion materials and internal evaluation reports (*n* = 27), (iii) participant observation at training and development events (*n* = 5) and (iv) ongoing email discussion with key players in the four sites over a 6-month period. Detailed interview and observation notes were recorded by the lead, second and third author in a narrative style with key verbatim comments being recorded where possible. Key themes were extracted from documents using a standard template. Data were integrated by extracting key themes against an analytical framework (which covered the following areas: history and drivers; community-level activity; genetic service activity; healthcare professional activity; leadership, management and partnerships; resources; roles; issues arising) as suggested by Ritchie and Spencer (1994). Commonalities, differences and cross-cutting themes across and within the sites were identified. A draft write-up of findings was circulated to respondents from all the sites for their additions, comments and corrections. In phase two, at the end of 2013, almost 2 years after the initial fieldwork, a follow-up deliberative workshop was convened involving 21 participants, in which three of the four original sites were represented, along with a further three sites where initiatives had been started in the intervening period. Email correspondence provided an update for the one original site that was not represented at the workshop. The workshop provided the opportunity for staff from the original sites to update the description of their current service offer, for newly initiated activities and resources to be shared both from the original and the new sites, and for the whole group to reflect upon both changes over time and persistent issues in this field of practice. Finally, in phase three, a further deliberative workshop was convened in April 2015. This involved representatives from two of the original sites, together with individuals involved in the development of service responses in a further four sites (two of which had been represented at the earlier, phase two workshop), with a total of 13 participants. Again, the workshop involved updates on service activity and structured discussion around key issues. Detailed, structured notes were taken in both workshops and key themes integrated with the earlier template. Gaining this longitudinal picture was important since a key issue raised during the initial fieldwork period was the questionable sustainability of the initiatives. Inclusion of data from the additional sites in phases two and three was useful in identifying issues that recurred across settings as well as those that reflected local context. While it should be acknowledged that our data generation was dominated by professional perspectives, some insight from community members and affected individuals was gained via reviewing material that had been generated in the sites via community consultation exercises and internal evaluation exercises (e.g. patient stories); indirectly via professional respondent reports of interactions with affected parents and local people; and workshop contributions from individuals who identified themselves as members of affected communities. Data generation during all the phases was focused on describing the services/activities and examining their implementation, guided by the following broad questions:

- How are service objectives framed?
- What do the components of the services look like?
- Which partners and stakeholders are engaged and in what ways?
- How is performance assessed?
- What challenges have been faced?
- What factors have supported or undermined sustainability?

## Findings

We first describe the service components across the four original sites at the initial review time point in 2011/2012 (see Table 1), organised around the three areas of WHO-recommended activity identified above. We highlight commonalities and differences and issues that arose in the design and delivery of services. We then turn to describe changes over time in the service offers across the four sites, as well as issues cross-cutting original and newly identified sites, highlighting factors that appear to have supported or undermined service development and sustainability in this area of practice.

**Table 1:** Overview of service responses across three strands of intervention in the four sites in 2011/2

Site	Stated objective(s)/outcome	Enhanced family-centred genetic counselling and testing	Enhancing community genetic literacy	Enhancing confidence and competence of health and other professionals
1 Population: 148,000 31 % Black and minority ethnic	To improve take up of genetic services by proactive targeting towards families affected by known autosomal recessive disorders	Part-time specialist genetic counsellor. Pilot intervention evaluated. Retrospective review of cases and subsequently prospective referral in. Home visits offered as well as clinic. Cascading to family members. Recent increase in resource to allow recruitment of additional team member to support counsellor and avoid reliance on one worker alone.	No activity to date. Future activity being considered.	Educational sessions run by the specialist genetic counsellor for a variety of health professionals. Including paediatricians, midwives, health visitors and GPs.
2 Population: 200,000 36 % Black and minority ethnic	Families at greatest risk of inheriting complex disorders are more informed about their risks and are using the services available to them	No enhanced activity to date.	One-year community engagement programme using a hub and spokes model. Community development workers trained and supported to deliver 1-to-1 and group sessions with service users and colleagues. Training booklets used and retained by service users to share with family. Signposting to GPs. Workshops at mosques. Dialogue with Imams. Inclusion of messages within widely distributed general infant mortality leaflet.	One-day training course delivered several times by external consultant.
3 Population: 270,000 23 % Black and minority ethnic	To reduce infant and perinatal mortality among societies with a preference for cousin marriage linked to high prevalence of recessive genetic disorders.	Part-time community genetic counsellor. Retrospective review of genetic case notes to identify families. Prospective referral from health professionals established; 1-to-1 counselling offered, home visits and outreach sessions. Cascading to family members.	Community events held in mosques and children’s centres using external consultant. Live channel broadcast in GP surgeries. Genetic counsellor runs awareness sessions at community venues.	A series of two-day training events delivered by external consultant.
4 Population: 300,000 70 % Black and minority ethnic	To offer improved reproductive choices by enhancing the awareness of and access to genetic services by Black and ethnic minority groups, particularly the Pakistani community.	Recruitment of specialist community genetic counsellor. Retrospective review of genetic case notes and re-contact. Home visits offered as well as clinic. Cascading to family members. Establishment of support groups for patients and carers living with conditions. Development of new molecular tests for a range of locally found conditions.	Community educator uses a range of approaches to engage local people e.g. mosques and children’s centres. Genetics community advisory group established to provide service feedback. Educational leaflets and DVD in English and Urdu. DVD posted on YouTube. Website created and operational.	Appointment of an educator in clinical genetics to provide education for all health professionals wishing to enhance skills: seminars, workshops, resources, support in clinical practice.

### Initial service components

#### Family-centred genetic counselling and testing services

Genetic counselling and testing services were provided in all the four original sites through the Regional Clinical Genetic Services (RGS) throughout the review period. However, at the time of our initial review exercise, three of the four sites had introduced an enhanced family-centred service (see Table 1), by increasing the capacity within the RGS through recruitment of a dedicated counselling worker.

A dedicated worker was felt to be valuable in building trust with the local community and encouraging service uptake. There were mixed opinions, however, as to what constitutes the necessary attributes and skills for this role. While some regarded community language ability as essential, others felt that interpreters are readily available. Similarly, some respondents emphasised the importance of religio-ethnic congruence, while others felt that achieving cultural competence is not necessarily linked to ethnic or religious identity. Respondents noted the possible downsides of being perceived by patients as a community insider including potential worries about lack of confidentiality. Some respondents also highlighted the need for services to be accessible to people of all ethnic identities, and therefore, that an ethnically diverse staff group was preferable, though difficult to achieve given the small numbers of staff involved.

Respondents in all the three sites acknowledged that recruitment was difficult, particularly if both genetics knowledge and cultural awareness were demanded. In one site, respondents told us, and an internal evaluation document reported that, progress had been much slower than planned due to the lack of a consistent worker. In another, a flexible approach had been taken involving the up-skilling of a worker who was not genetically qualified upon recruitment.

The specialist workers at all the three sites were based in hospitals. They initially used retrospective case reviews of index cases to identify parents who might benefit from the enhanced offer. They also had the flexibility to work from community facilities and conduct home visits, an approach that was felt to engage and empower family members and open up possibilities for information cascade. Home visits were, however, identified as costly. At the time of initial fieldwork, all the three sites were in the process of establishing prospective referral mechanisms from other health professionals (e.g. midwives, paediatricians, metabolic specialists) to address the issue of under-referral of eligible patients from other services. Co-location with antenatal care was considered helpful in ensuring quick referrals and had been implemented in one site.

All the sites reported offering cascade counselling to siblings in the first instance, and that decisions to proactively cascade information to wider family members were made on a case-by-case basis depending on the condition, diagnosis confirmation, availability of testing and the nature of the family structure. Cascading the offer was recognised as a major challenge. Inconsistent recording of consanguinity and ethnicity, a lack of computerised information and data protection concerns complicated the identification of eligible individuals. Stigma and isolation linked to living with disability were also factors identified as limiting opportunities to negotiate access to wider family members via index cases (the current norm within English genetic services). In the words of respondents from two different sites: “In practice it is very difficult to engage these families. The extended family are often difficult to access and if you do access them they are usually difficult to engage” and “There is huge reluctance to refer on other family members”*.* Transnationally dispersed families were noted as presenting additional barriers. Respondents also highlighted the need for more consistent practice in relation to longer-term recall of children to ensure an offer of genetic counselling in the future.

Success in all the three sites was largely judged in terms of whether the proactive offer of service was being taken up by individuals. Notwithstanding the common difficulty in generating cascade contacts, there was noticeable variability across the sites in reports of success in generating new service uptake, with two reporting good uptake and the third reporting disappointingly low uptake. While contextual differences are likely to play a part, respondents felt that the success of the enhanced offer is heavily dependent upon the specialist worker’s ability to engage with and win the trust of the local community. This raised questions about training, support and integration provided to these specialist workers if they work in isolation rather than as part of an established genetic team (as discussed more below).

All the sites reported generally very positive feedback among those individuals who had taken up the enhanced service offer, even where no genetic test could be offered (which remains the case for a sizeable proportion of rare conditions). Nevertheless, respondents noted the challenges of communicating complex genetic information to patients who often had limited formal education. No bespoke educational materials for this area of work were in use at the start of our review period, with workers relying instead on their own techniques of verbal explanation employing a mixture of community languages, English technical terms and culturally resonant metaphors. There were, however, useful developments over time in both print- and web-based patient information tailored to the needs of families at risk of recessive conditions linked to consanguinity (as discussed more below). One site also invested considerable resource in the development of new molecular genetic tests for rare conditions affecting their local population, while another took advantage of ongoing research studies to maximise mutation identification.

### Enhancing community genetic literacy

At the time of the initial fieldwork, three of the four sites reported investments in initiatives aimed at raising awareness and enhancing genetic literacy among members of the public (Table 1). Respondents from the fourth site expressed doubts as to the value of community-level activity directed at individuals with no known risk of a recessive condition. They questioned the likely success of raising general genetics understanding (“an ambitious task”) and expressed concern that there was “a danger of misinterpretation of messages among community members” (Fieldnotes, interview, 5/1/2012). There was great variety in the approaches adopted across the three sites where work was underway at community level, with differences apparent in engagement of individuals identified to be ‘local community leaders’; bearers of the information; content and framing of messages; and mode of delivery.

Establishment of a community steering/advisory group with representation from respected local people of Pakistani heritage (such as the director of a community-based organisation or a local politician) was felt to have been a useful approach in two sites, but it had not been easy to find people who were willing to align themselves with the agenda. There was also uncertainty over whether and how to engage religious scholars and leaders. While one site reported ‘fantastic’ responses from Imams they had approached, respondents at another site reported that they had decided not to engage religious leaders directly in service developments, anticipating the potential for misinformation. Nevertheless, across all the sites, supporting existing patients of the genetic service to seek religious counselling, if they so wished, was recognised as important.

Respondents highlighted the significant challenges in communicating complex information regarding individual- and population-level risk whilst also remaining sensitive to socio-cultural context. The review identified pros and cons of employing generalist versus specialist staff to deliver information at community level (Table 2). The review indicated that integrating information exchange into pre-existing community-level initiatives (such as drop in mother-and-baby sessions at Children’s Centres) reached greater numbers of people than putting on special events which were often poorly attended. Familiar faces and repeated contact seemed most likely to positively inform community conversations. In the two sites, we were informed of a legacy of past media damage and past unhelpful intervention. In one of these sites, the respondent told us that the current genetic counsellor was finding it very difficult to engage at community level and that there was a sense among some local people that the service was “unwelcome”:

[Fieldnotes, interview, 9/1/2012] Respondent reports that in the past there was damaging work in [name of site]. “Professionals have gone about it the wrong way. The wording [of messages] was unhelpful; challenging culture and religion. We have a duty to let people know of the services and tests while also making it clear that they have the right to marry who they want. We need to take a clear equalities perspective on this. [When I was talking with community members] they thought I was there to simply tell them not to marry their cousins. They were shocked when I said that I had come to talk about the service gap”.

**Table 2:** Approaches to enhancing community genetic literacy

Approach	Benefits	Challenges
Specialist staff outreach delivery (one-to-one or group sessions; with or without materials)	• Consistent and accurate message • Information tailored to need • Easy service referral if needed • Use of accessible community venues	• Limited reach • May be mistrusted • Anonymity not guaranteed • High cost (particularly if external consultants used)
Community-based existing generalist staff delivery (one-to-one or group sessions, with or without materials)	• Trusted by community • Sensitive to community dynamics • May encourage self-referral • Use of accessible community venues	• Require support • Danger of inaccurate and inconsistent messages • No tailoring of information to need • Competing agendas—focus lost • Anonymity not guaranteed
Stand-alone printed leaflets for general population (English and community languages)	• Wide reach • Anonymous access possible • Low cost	• Poorly targeted • Limited detail possible • Inaccessible to non-literate • No tailoring of information to need • No opportunity for clarification • Reliant on self-referral • Limited durability
Stand-alone printed booklets for families at risk	• Comprehensive information • Durable—repeated reading possible	• Limited reach • Reliant on self-referral • No opportunity for clarification • Inaccessible to non-literate • High cost
Stand-alone website material (including embedded video material)	• Easily accessed • Comprehensive information • Wide potential reach • Durable; repeat reading possible • Video content may be accessible to non-literate	• Unsuitable for some sub-groups • Reliant on self-referral • No opportunity for clarification
TV broadcast in GP practices	• Wide reach • Credibility of NHS location • Consistent, accurate • Durable; repeat watching possible	• Poorly targeted • Easily ignored • No opportunity for clarification • Reliant on self-referral • High cost
Integrated message within general infant health education materials	• Non-stigmatising • Wide reach • Anonymous access possible • Low cost	• Poorly targeted • Very limited detail • No tailoring of information to need • No opportunity for clarification • Reliant on self-referral • Limited durability

In the other site, a respondent emphasised the importance of working closely with the local community:

[Fieldnotes, interview, 12/12/2011]. Respondent reports that involvement of the community is key to building their trust. “The golden rule is don’t anger the community that you are targeting”.

Respondents across the sites acknowledged that it had proved difficult at times to ensure messages did not become inaccurate or stigmatising. For instance, there were concerns that the level of increased genetic risk associated with consanguineous marriage could be over-stated and that an overly simplistic message—that people should not marry within the family—was at times delivered. These experiences underscored the need for systems to quality control the flow of information, particularly if delivered by non-specialist staff and in a number of community languages.

All the sites confirmed that their primary message to anyone concerned that they or family members might be at risk was to seek professional advice, either from their family physician (general practitioner (GP)) or, where available, from the dedicated worker within the genetic service. However, there was great variation in terms of the detail and framing of the information provided to community members. An area of discussion related to the desire for materials to be readily accessed by those in need of information, while avoiding stigmatisation of particular communities. Looking across the materials that were in use, we found differing approaches in relation to the use of community languages, multi-ethnic versus ethnic-specific images, and messages embedded within general health materials versus dedicated resources. Other challenges included high costs of some materials, inaccessibility of written information to some community members whether in English or community languages, and the potential for misinterpretation of images and text in stand-alone materials not accompanied by verbal explanation (Table 2).

[Fieldnotes, interview, 12/12/2011] We discussed different materials and leaflets that are available with the respondents. They found some to be good at explaining genetics to professionals but decided cost was prohibitive. Materials needed to be even simpler and briefer for community members – they have found some confusion with text and pictures. “Simplicity is everything”. Some detailed booklets only really work if you can take the time to go through it in detail with the individual/family. So, if further information is required they would rather it was given face-to-face. They have produced a range of brief and simple resources for people who may need help in seeking support – but the materials are not designed to explain the issues – just to guide people towards professionals who can help.

Despite an acknowledgement of some initial suspicion and hostility from some local people, data from the three sites indicated that individuals who had engaged in face-to-face information sessions had welcomed these opportunities and largely gone away feeling better informed, often with misconceptions usefully clarified. These experiences therefore indicated that if the topic is approached in a sensitive way, many local people are responsive. The fourth site, however, reported less success in engaging at community level.

[Fieldnotes, observation of network meeting, 1/12/2011] Meeting participants note that there is an element of mistrust at community level due to prior negative messages delivered in relation to cousin marriage. However, considering the number of people who have attended group sessions (around 200) and the number of people who requested individual one-to-one consultations (over 60), it appears that people are interested in learning about the issue. Some people were concerned that the message delivered might clash with people’s religious beliefs. However, it is reported here that the involvement of local religious scholars (Mufti, Imam) has helped in this regard.

[Fieldnotes, interview, 9/1/2012] Respondent feels that in [site name] they have not managed the community development angle well so far. Respondent says that they tried to hold community events but then they heard of men telling their wives not to attend the community events - mistrust. They ran six half-day sessions, but these were not very successful in attracting people, very low attendance.

At the same time, the reach of the community genetic literacy activities appeared limited in all the sites. In one site, a review of the process evaluation report and monitoring data indicated that the involvement of men and younger people had been particularly low, with men making up just 17 % of beneficiaries. Reports from the other sites suggested a similar picture, and respondents highlighted the need for more differentiated approaches that respond to diversity within and between communities. None of the sites had undertaken any kind of baseline assessment of levels of knowledge and behaviours prior to community-level activity.

By the time of our 2013 workshop, some of the above concerns were being addressed in both the original and the newer service sites. For instance, web-based materials had been developed by one of the new sites, and interventions in colleges were being undertaken in another new site. Nevertheless, outstanding issues remained. Differing opinions as to what information should be delivered at community level were reflected in our review of the materials in use across the six sites that participated in the workshop, with significant variation persisting in content as well as in how the material was being distributed (i.e. stand-alone or with facilitation). A persistent concern was the potentially dangerous blurring of the line between general awareness-raising and genetic counselling related to individual circumstances, suggesting an ongoing need for clarification of professional boundaries for the various cadres of staff involved in delivering genetic information.

[Fieldnotes, interview, 12/12/2011] The respondent discussed the boundary between educator and counsellor and how to approach this issue when discussing with a family in the community. They have a clear line in [name of site] that whenever ‘advice’ is given that is from a clinical professional and if someone asks for advice from a community level worker then the questions are taken away to be answered and an appointment with a genetic counsellor is booked.

Concern to avoid inadvertent stigmatisation of minority ethnic communities was another common theme at the phase two workshop, though more prominent in some sites than others. This had led to one site turning down an opportunity to use local radio and another of the original sites to stop the roll out of a planned school-based educational programme. However, workshop representatives from another of the original sites reported in 2013 that the “initial level of suspicion did not persist, members of the community valued the service” and that “[it is an] emotive issue, but if dealt with sensitively conveying accurate risks, need not be controversial”*.*

#### Engaging and equipping the wider healthcare workforce

In 2011/2012, we found that all the four sites had invested in some activity aimed at engaging and equipping the wider healthcare workforce to support this agenda. Activity had primarily consisted of face-to-face training events, in recognition of the low levels of understanding among healthcare professionals (confirmed in two cases by an initial audit, as reported during field visits). Two of the four sites had called on an external consultant to provide the training, while the other two had used internal staff. Our review of curricula and participant observation suggested that session content was similar, covering inheritance patterns and genetic risk, socio-cultural context and broader issues related to professional boundaries and working with diverse patient populations. A key aim across the sites was to equip healthcare professionals with an accurate picture of the levels of increased risk and a nuanced understanding of the links between consanguineous marriage, recessive inheritance and congenital disorders. Though training was not mandatory in any of the sites, take up was reported to be generally good. There was, however, mixed success in engaging primary care GPs in these training initiatives. There were also concerns that healthcare professionals who chose not to attend might be particularly poorly informed and likely to convey unhelpful information to patients and the public.

Feedback on training from participants was reported to be positive both in interviews and in the two evaluation reports that we examined. However, at the time of our initial review, there had been no evaluation of the impact on knowledge or practice either in the short or longer term. Further, our review of curricula and participant observation at one training event found that the training did not always set out clearly what different cadres of healthcare practitioners were expected to do in their day-to-day practice in relation to the issue. In particular, there was lack of clarity regarding whether and when practitioners should proactively raise the issue with patients (rather than responding to patient queries); an issue that remained unresolved by the time of our follow-up workshop in 2013.

Other engagement activity with healthcare professionals had aimed at establishing referral mechanisms and opportunities for community awareness-raising within health service settings. Primary care centres had been a particular focus for these efforts. Respondents noted that GPs were potentially aware of families that would not necessarily come to light through genetic or paediatric services, such as those experiencing multiple early infant deaths. GPs were also intended to be the first port-of-call for individuals in the site without an enhanced genetic service. It was therefore considered important that GPs and other primary care staff provide a consistent and supportive message. However, at the time of our phase one data collection, respondents reported no success in getting GPs to actively refer patients who might benefit from the enhanced genetic counselling offer, not even in one site where an explicit payment system had been piloted. Some respondents reported that GPs in their area were known to provide inaccurate information and/or refuse to refer individuals who requested access to genetic services in relation to this issue. Reasons offered for the low GP engagement included low awareness of the issue (e.g. site 4 knowledge audit data), multiple competing demands, and a general push to reduce referrals into secondary care. Given that community-level awareness-raising activity conveyed the message to seek professional advice in case of concerns about a genetic condition within the family, the poor engagement of GPs was a concern, particularly where there had been no appointment of specialist genetic service staff. All the respondents highlighted the need for better engagement of GPs and primary care in the agenda; an issue that showed only limited improvement over time (as discussed more below).

[Fieldnotes, interview, 5/1/2012] The respondent reported that they have recently started trying to link up with GPs working in areas of high Pakistani concentration. The main aim here is to educate GPs and other practice staff so that they might support the accessing of extended family members for counselling and testing. GPs may also be aware of families where deaths/still births are linked to consanguinity. GPs are relevant to the bigger picture. Some evidence of GPs not referring to genetics some people who they deem not to be at risk when in fact they are extended family members of an affected family – need to address this in the context of GPs being encouraged to ‘manage demand’ for referrals to save costs.

[Fieldnotes, interview, 9/1/2012]) Respondent says that they had wanted them [GPs] to do a quick family tree and make a referral but they said that this would take an extra 7 minutes on each appointment and they therefore refused to do it without additional payments.

### Developments over time

Table 3 summarises the changes over time in the service elements being delivered across the four original service sites, and illustrates the sharply contrasting patterns found. We found evidence of innovation and service development throughout the period in site 1. Furthermore, our follow-up workshops drew in participants from further afield most of whom represented sites where services were only recently being developed, illustrating that this area of practice was emerging in other parts of the country over the period of the review. Meanwhile, in site 2, the service offer stagnated over the review period. In site 3, we found that interventions had been decommissioned by the time of the 2013 workshop. In site 4, interventions were withdrawn by 2015, despite substantial investment and ongoing development work during the first half of our review period. These findings indicate the vulnerability of this area of service development and raise questions regarding the factors that support or undermine both the innovation and sustainability of such initiatives.

**Table 3:** Change over time in service offer across original case study sites

Site	2011/2012			2013			2015		
	Family-centred counselling and testing	Community genetic literacy	Health professionals capacity development	Family-centred counselling and testing	Community genetic literacy	Health professionals capacity development	Family-centred counselling and testing	Community genetic literacy	Health professionals capacity development
1	✓	X	✓	↔	✓	↑	↑	↑	↑
2	X	✓	✓	X	↔	↔	X	↓	X
3	✓	✓	✓	X	X	X	X	X	X
4	✓	✓	✓	↑	↔	↔	X	X	X

✓activity underway/initiated; ↑ activity expanded; ↓ activity contracted; ↔activity continued; *X* no activity/activity ceased

#### Innovation and service improvement

The formative review identified three important cross-cutting issues that shaped service innovation and improvement over the time period: coordination across the three WHO strands; development of an evaluative culture; and engagement with a ‘Community of Practice’ (that is, a group whose members regularly engage in sharing and learning based around a common area of work (Lesser and Storck 2001)) .

We found variation across the sites in the intervention packages introduced, but findings tended to suggest the importance of coordinated action across all the three recommended areas. In site 1, the introduction of community-level activity in the form of two outreach workers part-way through our review period was notable both for its coordination by the specialist genetic counsellor based at the RGS and for its provision of follow-up support to patients who had been seen in clinic as well as general community education. Complementing the well-established enhanced genetic service offer with work to raise community genetic literacy seems likely to support engagement of extended family members. In contrast, work at community level without complementary improvement in GP engagement and enhancement to the genetic service offer—as in site 2—may fail to generate uptake of services that remain socio-culturally and physically distant from potential users. Equipping and engaging the wider health and social care workforce was consistently felt to be important, and there had been some notable improvements over time. For example, new materials, clearer guidance on professional roles and follow-up of trainees to assess subsequent practice were positive developments reported for site 1 during a presentation at workshop 3. Similar developments were also reported at the workshops by participants from some of the newly established sites. This area was, however, challenging, particularly in relation to primary care and GPs. Obstacles discussed at the workshops included competing priorities, assumptions that this issue is already dealt with elsewhere, and concerns about ability to provide adequate information. In the absence of GP buy in, there is a danger that individuals who seek advice may not receive an appropriate response or a timely referral. The importance of continued efforts at education and coordination was highlighted by persistently low levels of referrals into the enhanced genetic offer from other services/clinics across most sites. Over time, site 1 had made progress in establishing strong professional networks and referral procedures, and these were beginning to reap some referrals from general practice and antenatal services towards the end of our review period.

Another promising development over time in some sites was the increasing effort to evaluate and refine the service models on offer, including some evidence of the active involvement of affected individuals and members of the public in shaping responses. Two sites made significant progress both in terms of mapping the range of autosomal recessive conditions in their local populations and monitoring service uptake and patient journeys, as illustrated in data that were presented at the workshops. There were also useful attempts to gain input from community members to the development and evaluation of service approaches. For example, in one site, a leaflet aimed at supporting patients to share genetic information with family members had been developed with input from affected patients. One site had undergone an external evaluation, and another was engaged in an ongoing process of internal evaluation, both of which involved gaining patient perspectives. Nevertheless, it was recognised that more rigorous evaluative research, and greater input from affected communities, was needed to fully understand processes, effectiveness and impact (as discussed more below).

[Documentation review excerpt] The report notes that a lack of community representation to provide guidance to [name of site] was an issue identified by some local stakeholders who felt that greater development of this involvement should have happened prior to initiating work.

Linked to the above, participation in a fledgling Community of Practice, involving exchange visits, workshops and email communication, appeared to have been beneficial for the two original sites where activity was sustained over time as well as the three further sites that were represented at the later workshops and where activity was initiated later on. Respondents from both the original sites and the newer ones reported very positively on having opportunities to share their work and discuss challenges, particularly important given the very small number of staff working on the issue in any one location. Site 1, given its sustained development over time, was an important contributor of learning to the Community of Practice, but exchanges of insight happened in many directions. It was apparent that issues raised at workshops had subsequently been addressed in service development in some localities, such as the need for health professional training to cover particular content and the need to clarify professional boundaries, roles and responsibilities within the community genetic services infrastructure. There was evidence of service specifications and communication materials being shared across at least five sites, but there were also some missed opportunities to pool resources and learning. Developing a strong Community of Practice, engaging the whole set of stakeholders, including affected parents and community representatives, was felt to be important, but it was less clear how this would be sustained in practice given the limited budgets and small number of staff involved. Respondents felt that national bodies, such as the National Genetics Education and Development Centre and Public Health England, could do more to support this area of practice.

#### Sustainability

Our review highlighted three inter-related factors that appeared to affect the sustainability of these service innovations: alignment of commissioner expectations with service realities; integration of service developments within the mainstream offer; and champions.

The challenge of securing sustained investment for service developments was raised as an issue in our initial review phase and became more prominent at the follow-up workshops. Securing resources was closely linked to the challenges of demonstrating success. Furthermore, it appeared that in some sites, those commissioning the service had unrealistic expectations regarding both the level of service uptake/engagement and the timeframe within which impacts might be seen. In site 3, at the time of our initial review work, a respondent identified the challenge of justifying investment and demonstrating impact:

[Fieldnotes, interview, 9/1/2012] Respondent reports that a lack of funding is an issue and organisational change has not helped. Impact will not show for 5 years or so, so in the meantime it is a challenge to show evidence of its value. They are struggling to identify performance indicators for the work; “How can we monitor and show we are effective?”

In site 4, divergent expectations between commissioners and providers, regarding patient numbers and reductions in infant mortality and childhood disability at population level in the short to medium term, were highlighted by respondents and also in the evaluation report we reviewed (Document review notes: unrealistic timeframe expectations to reduce infant mortality; time for family decision making not adequately recognised by commissioners). The consensus at the 2013 deliberative workshop was that this area of work requires patience and long-term investment, since individuals and families often take time to comprehend the new information being given to them and to engage with services or shift reproductive behaviour. Furthermore, participants emphasised the difficulty of assessing whether both patterns of service uptake (or non-uptake) and subsequent reproductive behaviour reflect informed decision making or not. It was clear that unless key decision-makers recognise this reality and identify appropriate performance indicators, investments are vulnerable.

A further factor that appeared to be important in sustaining activity was the degree of integration of the new innovations within pre-existing service structures. Integration related both to structural arrangements and to professional relationships and attitudes. Thus, in sites 3 and 4, the enhanced genetics offer was generally perceived to be a stand-alone ‘project’. In contrast, in site 1, the investment seemed to be viewed as increased resource for the genetic service itself, providing a welcome opportunity to address unmet need. Meanwhile, in site 2, educational activities with community members and healthcare professionals had largely taken place without engagement of the Regional Genetic Service. Limited integration with the core service in site 4 meant that problems around personnel recruitment, retention and support were not easily addressed. In sites 3 and 4, the specialist workers struggled to encourage appropriate referrals from genetic service colleagues, with knock on effects for the level of service activity. In contrast, in site 1, the specialist worker appeared to have been well supported and embedded within the core work of the genetics unit from the early days of service development. Given the heavy reliance on very small numbers of staff to develop and deliver the new services across all the sites, integration with pre-existing services may also help to retain organisational knowledge in the face of staff turn-over.

The importance of having dynamic individuals championing the work was also clearly important to sustainability. In site 3, which had decommissioned its work by 2013, the original commissioning manager had moved to a new job outside the area, and there was little by way of community engagement in the initiatives. In site 2, where stagnating activity was evident in 2015, the lack of a champion with a clearly designated remit for this work was identified by local representatives at our deliberative workshop as a major obstacle to sustaining progress. Similarly, a lack of champions was identified in site 4 as a factor that undermined securing renewed resources for the project. In contrast, the ongoing commitment and creativity of a public health consultant had clearly been instrumental in retaining, and increasing, investment despite changing commissioning structures in site 1. Furthermore, with a backdrop of increasing clinical demand and reductions in other areas of service, close involvement of senior genetic service staff in site 1 seems likely to have been important to securing continued activity in this area.

## Discussion and conclusion

We recognise that the findings reported here are primarily informed by professional perspectives on the issue and that additional work to understand more about patient and public understandings and experiences of services is needed to provide a comprehensive picture. Furthermore, rigorous, outcome evaluations will be needed to assess effectiveness and cost-effectiveness. Nevertheless, our formative service review provides important insight into a rapidly evolving area of practice, generating early understanding of key components and processes of service activity.

A number of cautious conclusions can be drawn. First, in relation to family-centred genetic services, a targeted offer of culturally competent genetic counselling and testing to consanguineous parents with a child affected by a recessive disorder does appear to address past service gaps. Experience to date suggests that most individuals in this situation welcome the service offer and feel benefited by the new knowledge they gain. It is also clear that, contrary to common assumptions, individuals of minority ethnic and religious identity may opt for testing and may modify their reproductive behaviour in response to new information (Khan et al. 2010; Shaw 2015). Cascading genetic counselling and testing to extended family members of an affected individual is clearly more challenging, with individuals requiring significant support to share information with relatives. Our review suggested mixed success in this area as well as varied opinions among professionals regarding the degree of effort that should be invested here, perhaps reflecting the lack of national guidance in this area. However, recent research from Darr et al. (2015) suggests that communication among family members may be supported by new communication tools provided timely and extended consultations are available. Second, in relation to community genetic literacy initiatives, our findings suggest that despite suspicion and hostility from some quarters, when the topic is approached sensitively, such initiatives are received positively by many people. Nevertheless, their scale to date has been limited, and their impact on knowledge, behaviour and uptake of services remains uncertain. Conveying complex information is challenging and maintaining trust at community level remains a key concern for many commissioners and practitioners engaged in this area of work. Third, in relation to equipping the wider workforce, engaging healthcare professionals, particularly in primary care, is considered important but is often difficult to achieve in practice. While there have been some successful training events, more needs to be done to link these clearly to explicit service models, referral pathways and practitioner roles and to ensure sustained levels of knowledge and confidence (particularly for professionals who encounter this issue only intermittently).

The review highlights the great variation in current services/inputs provided across all the three strands of activity and suggests there would be benefit from more clearly articulated objectives and ‘intervention theory’ (Moore et al. 2008). In particular, there were mixed views among stakeholders regarding key elements of the community-level educational offer, including how messages should be framed, the degree of pro-activity versus responsiveness, whether and how places of worship and religious leaders should be engaged, and the degree of ethnic tailoring versus generic provision. It is also clear that local contextual factors will importantly shape the feasibility and appropriateness of different approaches, but the currently limited evidence makes it difficult to identify the factors that need to be taken into consideration. More rigorous evaluation, and more opportunities for open discussion and debate, will be important to reach consensus and agreed standards on service approaches. Such knowledge building is particularly important in service development aimed at meeting the needs of minority ethnic people since a preponderance of short-term, poorly documented initiatives have frequently contributed to a belief that ‘nothing works’ (Salway et al. 2013). Furthermore, more sustained and active involvement of patients and local people in the design and evaluation of initiatives should be a priority since this remained rather limited across all the sites.

The service review clearly demonstrates that sustaining investment was difficult, with just one out of the four initial sites maintaining momentum over the review period. Sustaining innovation in routine practice will often depend on a different set of factors than its initial uptake (Murray et al. 2010). Integration with mainstream services and having champions for the initiative appeared to be important here, findings that resonate with other areas of service development for minority ethnic populations (Salway et al. 2013). However, a more fundamental issue appeared to be inappropriate expectations regarding the impact of new services and activities. Thus, despite the commonly stated core goal of empowering informed reproductive decision making (see Table 1), a population health agenda, linked to national infant mortality targets and demands for cost savings in health and social care, was the dominant frame of reference in some sites. While it is plausible that the current modest levels of investment will be more than compensated in the long term by cost savings linked to affected births being averted, any such impact will take time. If investments in new activity and services are won entirely on the basis of business cases that argue in terms of reductions in infant mortality and childhood disability, they will remain vulnerable. Furthermore, a dominant population health perspective can alienate genetics professionals. Instead, it should be understood that this is a long-term process and that the guiding rationale must be one of tackling inequality and empowering individuals with information on which to make informed reproductive choices.

Many of the issues we have identified here point to the need for a national strategy and greater inter-regional collaboration (as already argued by Modell and Darr 2002). The growing number of independent service responses in different parts of the country makes this now an urgent priority. Without investment in evaluation and coordinated service development initiatives, resources will be used inefficiently, and benefits to individuals and families will not be maximised.
